# Development of Neutralizing and Non-neutralizing Antibodies Targeting Known and Novel Epitopes of TcdB of *Clostridioides difficile*

**DOI:** 10.3389/fmicb.2018.02908

**Published:** 2018-12-06

**Authors:** Viola Fühner, Philip Alexander Heine, Saskia Helmsing, Sebastian Goy, Jasmin Heidepriem, Felix F. Loeffler, Stefan Dübel, Ralf Gerhard, Michael Hust

**Affiliations:** ^1^Department Biotechnology, Institute for Biochemistry, Biotechnology and Bioinformatics, Technische Universität Braunschweig, Braunschweig, Germany; ^2^Institute for Toxicology, Hannover Medical School, Hannover, Germany; ^3^Department Synthetic Array Technologies, Max Planck Institute of Colloids and Interfaces, Potsdam, Germany

**Keywords:** Toxin B (TcdB), *Clostridioides difficile*, antibody phage display, recombinant antibody, epitope mapping, neutralization, scFv, scFv-Fc

## Abstract

*Clostridioides difficile* is the causative bacterium in 15–20% of all antibiotic associated diarrheas. The symptoms associated with *C. difficile* infection (CDI) are primarily induced by the two large exotoxins TcdA and TcdB. Both toxins enter target cells by receptor-mediated endocytosis. Although different toxin receptors have been identified, it is no valid therapeutic option to prevent receptor endocytosis. Therapeutics, such as neutralizing antibodies, directly targeting both toxins are in development. Interestingly, only the anti-TcdB antibody bezlotoxumab but not the anti-TcdA antibody actoxumab prevented recurrence of CDI in clinical trials. In this work, 31 human antibody fragments against TcdB were selected by antibody phage display from the human naive antibody gene libraries HAL9/10. These antibody fragments were further characterized by *in vitro* neutralization assays. The epitopes of the neutralizing and non-neutralizing antibody fragments were analyzed by domain mapping, TcdB fragment phage display, and peptide arrays, to identify neutralizing and non-neutralizing epitopes. A new neutralizing epitope within the glucosyltransferase domain of TcdB was identified, providing new insights into the relevance of different toxin regions in respect of neutralization and toxicity.

## Introduction

By the end of the 1970s, *Clostridioides* (former *Clostridium*) *difficile* (*CDiff*) was identified as the causative pathogen of antibiotic treatment associated diarrhea (CDAD) (Bartlett et al., 1978). Since then, the number of *CDiff* infections (CDI) has been increasing and in the last two decades *CDiff* even caused epidemic outbreaks (Rupnik et al., 2009; DePestel and Aronoff, 2013). In 2011, *CDiff* caused ~453,000 incident infections in the USA with ~29,000 deaths (Lessa et al., 2015). Due to its association with antibiotic treatment and the resulting high potential for development of antibiotic resistance, the Centers for Disease Control and Prevention (CDC) classify *CDiff* as an urgent threat (Centers of Disease Control Prevention, 2013).

In standard therapy for mild to moderate CDI, *CDiff* is targeted with metronidazole, vancomycin or fidaxomicin (Tedesco et al., 1978; Bolton and Culshaw, 1986; Goldstein et al., 2012). However, antibiotic therapy presumably further disrupts the gut microbiome that confers colonization resistance against *CDiff*. Hence, in 20–30% of CDI cases, recurrences or relapses occur within 2–6 weeks after completion of antibiotic treatment (Pépin et al., 2006). Another concern about antibiotic therapy is the high potential of *CDiff* to evolve resistances (Centers of Disease Control Prevention, 2013; Gao and Huang, 2015), therefore, alternative therapeutic approaches are urgently needed.

Disease and typical symptoms of CDI are only caused by strains that express at least Toxin B (TcdB), mostly together with Toxin A (TcdA) (Natarajan et al., 2013). Some strains also express an additional binary Toxin CDT, but its role in disease is still poorly understood (Gerding et al., 2014).

In the last two decades, the incidence of so-called hypervirulent *CDiff* strains has increased. These strains carry mutations within the toxin repressor gene tcdc, which may lead to higher toxin expression levels and, therefore, to more severe disease (Razavi et al., 2007; Joost et al., 2009).

**Figure 1:**
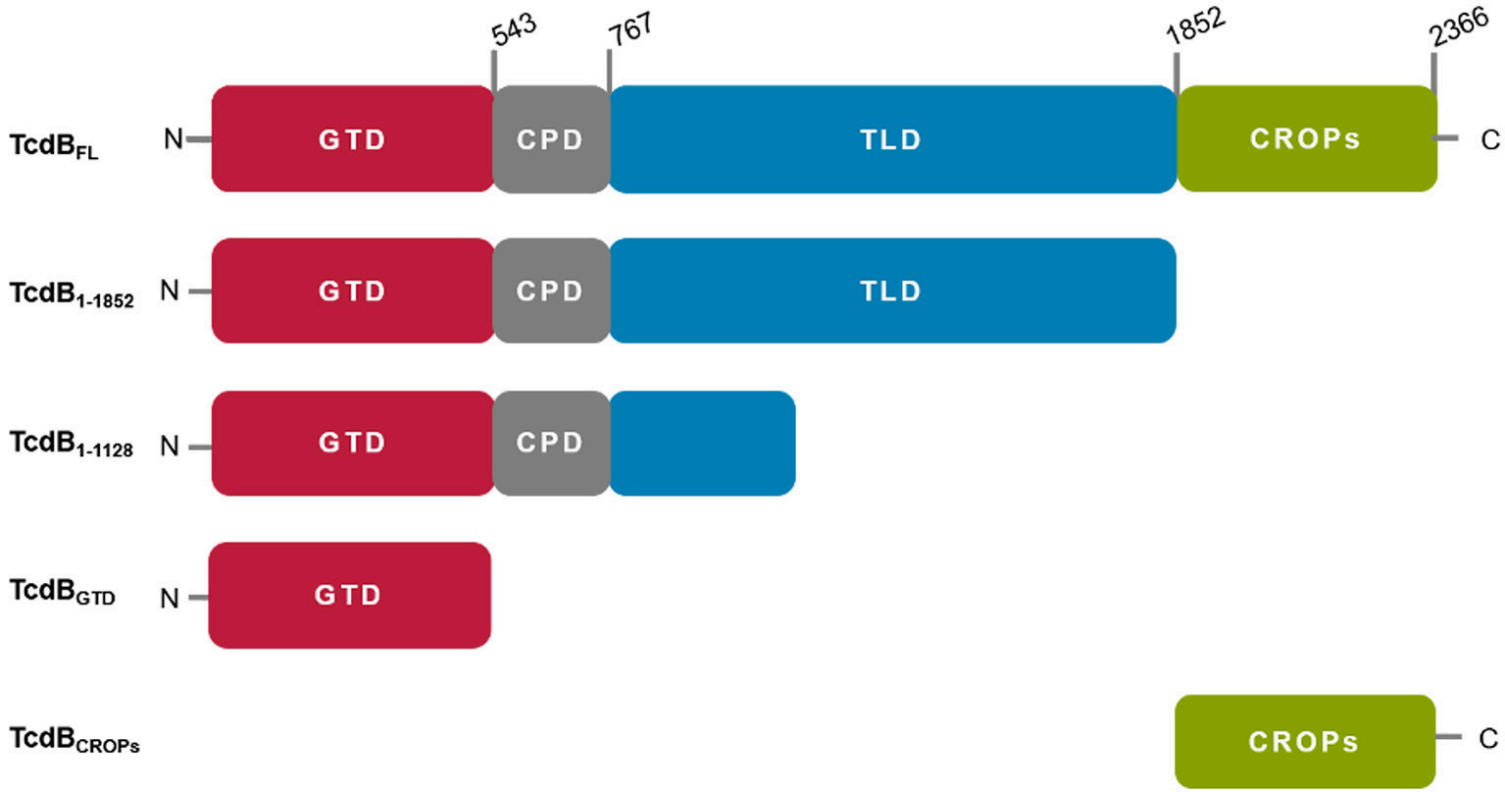
Schematic representation of TcdB fragments used in this study. All fragments were derived from TcdB of *C. difficile* strain VPI10463. TcdB_FL_: wild type (wt) TcdB, TcdB_1−1852_: wt TcdB missing the CROP domain, TcdB_1−1128_: N-terminal 1128 aa of wt TcdB, TcdB_GTD_: enzymatically inactive mutant (D286/288N) of TcdB glucosyltransferase domain, TcdB_CROPs_ combined repetitive oligopeptides, missing the first short repeat.

TcdA and TcdB are homologous single-chain multidomain proteins with a molecular weight of 308 and 270 kDa, respectively. A schematic representation of TcdB is given in Figure 1.

The N-terminal glucosyltransferase domain (GTD, TcdB aa 1–543) acts on small Rho-GTPases, e.g., RhoA, within the cytosol of the host's cells (Just et al., 1995; Busch et al., 1998). Due to the monoglucosylation, the GTPases are trapped in an inactive state, which inhibits multiple signal cascades, leading to cytoskeleton breakdown and consequently cell rounding (Rothman et al., 1984; Erdmann et al., 2017).

Amino acids 544–767 build up a cysteine protease domain (CPD) that catalyzes the proteolytic auto-processing and releases the GTD into the cytosol upon translocation, after activation induced by cytosolic inositol-6 phosphate (InsP6) (Egerer et al., 2007; Reineke et al., 2007; Shen et al., 2011).

Amino acids 768–1852 form the translocation domain (TLD). Despite notable progress during the last years, the exact function and the molecular mechanisms involving this huge domain are still elusive. The TLD includes a stretch of amino acids (aa 830–990), which are proposed to be involved in pore formation for translocation of the N-terminal portion across the endosome membrane upon acidification (Genisyuerek et al., 2011). Furthermore, for TcdB three putative receptors binding regions have been identified recently within this domain, which interact with the following cell surface receptors: chondroitin sulfate proteoglycan 4 (CSPG4), polio virus receptor like 3 (PVRL3) or members of the frizzled protein family (FZD1/2/7) (LaFrance et al., 2015; Yuan et al., 2015; Tao et al., 2016; Gupta et al., 2017). The role of these receptor binding sites in disease is still unknown.

The C-terminus of both toxins is composed of repetitive elements, where long and short repeats are combined in so-called CROPs (combined repetitive oligo peptides) (von Eichel-Streiber et al., 1992). In case of TcdA, the CROPs interact with carbohydrate structures [α-Gal-(1,3)-β-Gal-(1,4)-β-GlcNAc] on the cell surface of the target cells, mediating a first contact between toxin and target cell (Krivan et al., 1986; Greco et al., 2006) and prevent premature autoproteolytic cleavage of the toxin, by stabilization of the toxin conformation (Olling et al., 2014). For TcdB the role of the CROPs is less defined, but due to the homology similar functions can be assumed. Recently it had been shown, that next to residues within the TLD the first three short repeats of the CROPs are also involved in CSPG4 binding (Gupta et al., 2017).

Since TcdA and TcdB are the major virulence factors of *CDiff* responsible for damage of the gut epithelium upon CDI, efforts have been made to develop therapeutics that directly target the toxins instead of the bacterium (Kurtz et al., 2001; Puri et al., 2015; Sturino et al., 2015; Ivarsson et al., 2017). One of these is the human monoclonal anti-TcdB antibody Bezlotoxumab, recently approved by the FDA as therapeutic for prevention of recurrent CDI (U.S. Food and Drug Administration, 2016). However, this antibody only reduces the relapse rate by ~40% (Navalkele and Chopra, 2018) and is not approved for treatment of acute CDI. Crystal structure analysis revealed binding of Bezlotoxumab to two homologous epitopes within the CROPs domain (Orth et al., 2014). For either TcdA and TcdB it had been shown that toxicity is partially reduced, but not abolished in CROPs deletion mutants (Frisch et al., 2003; Olling et al., 2011; Manse and Baldwin, 2015), pointing out functionally independent receptor binding sites within at least TcdB, which could also affect neutralization efficacy of Bezlotoxumab. Therefore, a combined approach with a mixture of antibodies that target different epitopes or domains may further improve toxin neutralization and clinical outcome.

In this study, we describe the generation of a panel of human antibodies targeting different domains of TcdB by a phage display approach using the naïve human antibody libraries HAL9 and HAL10 (Kügler et al., 2015). In a cell based assay, the generated antibodies were screened for TcdB neutralization. Furthermore, domain and epitope mapping of the neutralizing and non-neutralizing antibodies was performed by antigen ELISA, peptide array (Weber et al., 2017a,b) and phage display. We mapped TcdB in respect to its epitopes, related to neutralization or, respectively, to non-neutralization. These resultsgive new insights into the relevance of different toxin regions regarding neutralization and toxicity. In addition, we identified a new epitope within the N-terminal glucosyltransferase domain (GTD) of TcdB that conveys neutralization.

## Methods

### Antigen Production

Full length TcdB from *Clostridioides difficile* strain VPI10463 (identical to TcdB from strain cdi630) as well as toxin fragments and isolated domains were recombinantly expressed as C-terminally 6 × His-tagged proteins in the *Bacillus megaterium* expression system (MoBiTec, Germany) (Burger et al., 2003), except for TcdB_CROP_ domain (aa 1853–2366) which was expressed as GST-fusion protein in *E. coli*. Full length TcdB_1−2366_ (TcdB_FL_), TcdB_1−1852_, TcdB_1−1128_, and TcdB_1−542_ (TcdB_GTD_) were cloned into pHIS1522 via *Bsr*GI and *Bam*HI (Wohlan et al., 2014). For production of TcdB_GTD_ we used the glucosyltransferase-deficient mutant TcdB_1−542_ D286/288N for higher yield and purity of protein. The His-tagged proteins were purified via Ni^2+^-affinity chromatography (Ni-IDA columns, Machery-Nagel, Germany) by gravity flow after protocol supplied by company. The purified proteins were stored at −80°C in storage buffer (50 mM NaCl, 20 mM Tris HCl, pH 8.0) after buffer exchange via Zeba desalting columns (Pierce, Thermofisher). The TcdB_CROP_ domain was expressed as N-terminal GST fusion protein. The fusion protein was purified via glutathione (GSH)-sepharose (GE Healthcare) after standard protocol in *E. coli* lysis buffer (20 mM Tris, pH 8.0, 50 mM NaCl, 1 mM dithiothreitol). TcdB_CROP_ was cleaved directly from GSH-sepharose bound GST-tag by thrombin at 4°C overnight. The purity and specific concentration of all proteins was estimated by SDS-PAGE.

### Antibody Generation

Antibodies against TcdB were selected in scFv-format from the human naïve antibody gene libraries HAL9 and HAL10 (Kügler et al., 2015). The selection and screening was performed as described before (Russo et al., 2018a). In brief, for antibody selection, scFv phage from HAL9 and HAL10 were mixed and incubated on TcdB_FL_, TcdB_1−1852_, or TcdB_CROPs_, immobilized in Costar High Binding microtiter plates (Sigma-Aldrich Chemie GmbH, Munich, Germany). Panning was performed at 37°C or room temperature. In two approaches, negative selection of the library on immobilized TcdB_1−1128_ was used to specifically isolate binders directed against the TLD domain of TcdB. After three rounds of panning, monoclonal soluble scFv were produced and screened for TcdB binding by antigen-ELISA. DNA of binding candidates was isolated and sequenced. The unique scFv sequences were recloned into pCSE2.6-hIgG1-Fc-XP (Beer et al., 2018; Russo et al., 2018b) using NcoI/NotI (NEB) for mammalian production as scFv-Fc, an IgG-like antibody format. The production in HEK293-6E cells and subsequent protein A purification was performed as described before (Jäger et al., 2013)

### Domain Mapping by Antigen ELISA

Costar High Binding microtiter plate were coated with 100 ng of TcdB fragments or full length TcdB (TcdB_FL_) in 100 μL of PBS overnight at 4°C. After saturating the wells with 250 μL MPBST (PBS with 0.05% (v/v) Tween20 and 2% (w/v) milk powder) and three times washing, a serial dilution of scFv-Fc in MPBST was added to the plate and incubated for 1 h at room temperature followed by three times washing. Bound scFv-Fcs were detected using a Fcγ specific HRP conjugated antibody (Sigma Aldrich, A0170, 1:70,000 in MPBST) for 45 min incubation at room temperature followed by 3x washing. The detection was performed using TMB substrate. The colorimetric reaction was stopped by addition of 100 μL 0.5 M H_2_SO_4_ and measured with an ELISA reader (TECAN Sunrise, 450 nm, reference 620 nm).

### SDS-PAGE and Immunoblot

TcdB was heated to 96°C in Leammli buffer with 3% 2–Mercaptoethanol for 10 min to denature. Five microgram of denatured TcdB was applied to an 10% SDS-PAGE and separated at 200 V for 45 min. The protein was transferred to a PVDF membrane using the Trans-Blot® Turbo™ (BioRad) transfer system according to manufacturer's instructions using the high molecular weight program (1.3 A, 25 V, 10 min). The membrane was blocked in MPBST and placed into a Mini-Protean®II multi-screen chamber (BioRad). The channels were completely filled with scFv-Fcs diluted in MPBST to a concentration of 1 μg/mL. After 1.5 h the channels were washed three times with PBST. The membrane was removed from the multi-screen chamber washed again with PBST and incubated with an anti-human IgG (Fcγ specific) AP conjugated antibody (Jackson Immuno Research Laboratories 109-055-098) in a 1:20,000 dilution in MPBST. After 1 h the membrane was washed 2 times with PBST and once with AP substrate buffer (100 mM Tris HCl pH 9.5, 0.5 mM MgCl2. Afterwards the immunoblot was developed with nitro blue tetrazolium chloride (0.30 mg/mL) and 5-Bromo-4-chloro-3-indolyl phosphate (0.15 mg/mL) in AP substrate buffer. The color reaction was stopped by removing the substrate through washing with water. The membrane was dried between paper towels and scanned.

### Cultivation of Vero Cells and *in vitro* TcdB Neutralization Assay

For *in vitro* TcdB neutralization assay a Vero cell line (African green monkey kidney cells) was used. TcdB treatment leads to a breakdown of the actin cytoskeleton which leads to cell rounding. This effect is easily visible using bright field microscopy.

Vero cells were cultivated in RPMI medium supplemented with 2.0 g/L NaHCO_3_, 2 mM stable glutamine (FG1215, Biochrom) and 10% fetal calf serum at 37°C and 5% (v/v) CO_2_ and passaged 2–3 times per week when confluency exceeded 90%. In an initial assay the working concentration of TcdB was determined. Therefore, Vero cells were seeded at a density of 10,000 cells per well in a 96 well cell culture plate (Cellstar®, Greiner bio-one) 16 h before intoxication, TcdB_FL_ was diluted in cultivation media and a serial dilution was prepared. Consumed media was removed from the cells, and TcdB_FL_ dilutions were added. Cells were incubated at 37°C and 5% CO_2_ for 5 h. Pictures of the wells were collected (Zeiss Axiovert 200 with Hamamatsu C4742-95 digital camera) and the percentage of round cells was determined by software assisted counting (Image J).

For neutralization assay, cells were prepared as described above and 0.1 pM TcdB_FL_ was premixed with scFv-Fc in cultivation media. After incubation of 30 min at room temperature the antibody TcdB_FL_ mixture was transferred to the cells. Pictures of the wells were collected when the percentage of round cells in the control wells (TcdB_FL_ w/o antibody) reached 70–80%.

### Construction and Packaging of TcdB-Fragment Phage Display Library

The pHORF-*tcdB*-fragment library was generated as described before (Zantow et al., 2016) with minor adjustments. In brief, *tcdB* was amplified from genomic DNA of *Clostridioides difficile* strain 630 (kindly provided by Meina Neumann-Schaal, DSMZ) by PCR using Phusion DNA polymerase and the following oligonucleotides as primers:

5′ATGAGTTTAGTTAATAGAAAACAGTTAGAAAAAATGG 3′ (forward),
5′CTATTCACTAATCACTAATTGAGCTGTATCAGG 3′ (reverse)

PCR product was fragmented using Bioruptor® Pico sonicator (Diagenode) using following settings: 4°C, 45 cycles, 30 s sonication (low intensity), 30 s pause. Fragmented DNA was concentrated using Amicon Ultra Centrifugal filters (30K MWCO, Millipore). Cohesive ends were blunted and blunt ends were phosphorylated according to manufacturer's instructions (Fast DNA End Repair Kit, Thermo Scientific). DNA product was purified using HiYield® Gel/PCR DNA fragment extraction kit, (SLG). A 10-fold molar excess of gene fragments was ligated into PmeI (NEB) linearized and CIP (Calf Instestine Phosphatase, NEB) dephosphorylated pHORF3 library vector (Kügler et al., 2008) (16 h at 16°C, T4 DNA Ligase, Promega). The ligase was inactivated at 65°C for 10 min and the buffer was exchanged to H_2_O using Amicon Ultra Centrifugal Filters (30K MWCO). Five microliter of ligation was used to transform 25 μL of electrocompetent *E. coli* TOP10F' (TOP10F' Electrocomp™ Kit, Life Technologies) by electroporation (1.8 kV, MicroPulser™, BioRad). Successfully transformed TOP10F' cells were selected on 2 × YT agar [1.6% (w/v) tryptone, 1% (w/v) yeast extract, 0.05% (w/v) NaCl, 1.2% (w/v) agar] supplemented with 100 mM glucose and 100 μg/mL ampicillin.

Determination of transformation rate and packaging of oligopeptide phage library and ORF enrichment was performed as described before (Zantow et al., 2016). Gene coverage and ORF enrichment was analyzed by sequencing of individual *E. coli* XL1 blue MRF' clones infected with TcdB-gene fragment phage.

### Epitope Mapping by Phage Display

Epitope mapping by phage display was either conducted by panning on scFv-Fc immobilized in Costar High Binding microtiter plates or as a panning in solution with immunoprecipitation using Protein A coupled magnetic beads (SureBeads, BioRad) with subsequent screening ELISA using monoclonal TcdB-fragment phage.

#### Panning on Immobilized scFv-Fc

For negative-selection of the TcdB-fragment phage display library, an irrelevant scFv-Fc (1 μg in 100 μL PBS/well) was immobilized in a Costar High Binding microtiter plate. After blocking with 250 μL panningblock [PBS with 0.05% (v/v) Tween20, 1% w/v BSA and 1% w/v milk powder] and 3x washing of the cavity using Tecan Columbus microplate washer, 10^9^ cfu TcdB-fragment-phage diluted in 150 μL panningblock were incubated in the negative-selection well for 1 h. The supernatant of the negative selection well and 1 μg of the irrelevant scFv-Fc for competition were transferred to the first of three successive panning wells, which had been coated overnight at 4°C with 1 μg of the scFv-Fc of interest and blocked with MPBST (PBS with 0.05% (v/v) Tween20, and 2% w/v milk powder). After 1 h of incubation, the phage were transferred to the second panning well, after one further hour to the third well. To prevent the first and the second well from drying, 1 μg of the irrelevant scFv-Fc diluted in 150 μL PBST was added immediately after transfer. To remove unbound phage, the first well was washed 10x with a harsh bottom wash program using Tecan Columbus microplate washer, the second well was washed 6x and the third well 3x using PBST. Remaining phage were eluted with 150 μL trypsin in PBS (10 μg /mL) by incubation at 37°C for 30 min. 10 μL of eluted phage were used to infect 50 μL *E. coli* XL1 blue MRF' at an O.D_600nm_ of 0.5. To generate single clones infected *E. coli* was plated on 2x YT-agar supplemented with 100 mM glucose and 100 μg/mL ampicillin and incubated overnight at 37°C.

#### Panning in Solution

For negative-selection of the TcdB-fragment phage library, a cavity of a Costar High Binding microtiter plate was coated with 1 μg of an irrelevant scFv-Fc in 100 μL PBS overnight at 4°C, then blocked with panningblock for 1 h at room temperature and subsequently washed 3x with PBST using a microplate washer. 10^9^ cfu TcdB-fragment-phage were diluted in 150 μL 2% (w/v) BSA in PBST and incubated in the negative-selection well for 1 h at room temperature. Magnetic protein A coated SureBeads (Bio-Rad) were washed according to manufacturer's instructions. Phage were transferred to a protein low binding (PLB) microtube (Sarstedt) and co-incubated with 15 μL resuspended beads for 1 h on a programmable rotator-mixer (PRM) (PTR-30 Grant-bio) to further remove sticky or unspecific phage. Beads were separated in a magnetic rack and phage containing supernatant was transferred to a fresh PLB microtube and co-incubated with 100 ng scFv-Fc diluted in 150 μL blocking buffer [2% (w/v) BSA in PBST] for 2 h on a PRM. An excess of magnetic protein A coated SureBeads (Bio-Rad) was added to precipitate the scFv-Fc fragments together with the bound TcdB-fragment phage (30 min, PRM). Beads were separated in a magnetic rack and the supernatant was discarded. Beads were washed 10x with 1 mL PBST, then resuspended in 150 μL trypsin (10 μg/mL) and incubated at 37°C for 30 min to elute the phage. Infection to obtain individual clones was performed as described before (Panning on immobilized scFv-Fc).

#### Production of Monoclonal TcdB-Fragment Phage and Screening ELISA

Each well of a 96 well polypropylene U-bottom plate was filled with 150 μL 2x YT-media [1.6% (w/v) tryptone, 1% (w/v) yeast extract, 0.05% (w/v) NaCl] supplemented with 100 mM glucose and 100 μg/mL ampicillin and inoculated with single *E. coli* colonies derived from the panning (see above) and incubated (37°C, 800 rpm, Labnet Vortemp 56) overnight. The plate was sealed with a breathable film (STARLAB INTERNATIONAL). For phage production 150 μL of fresh 2x YT-GA per well were inoculated with 10 μL overnight culture and incubated at 37°C and 800 rpm until cells reached exponential growth phase. Cells were infected with 20 MOI (multiplicity of infection) Hyperphage (M13K07Δ gIII) (Rondot et al., 2001; Soltes et al., 2007) for 30 min at 37°C without shaking and 30 min at 37°C at 800 rpm. After pelleting for 10 min at 3,220 × *g* cells were resuspended in phage production media (2x YT-media supplemented with 100 μg/mL ampicillin and 50 μg/mL kanamycin). Phage were produced during overnight incubation at 30°C and 800 rpm. Cells were pelleted at 3,220 × *g* for 10 min and phage containing supernatant was used for screening ELISA.

For screening ELISA, a Costar High Binding microtiter plate were coated with 100 ng scFv-Fc, respectively, 100 ng of irrelevant scFv-Fc as control in 100 μL PBS overnight at 4°C. Wells were saturated with MPBST for 1 h at room temperature The plates were washed 3x with water containing 0.05% (v/v) Tween20 before adding the monoclonal phage containing production supernatant (25 μL in 75 μL MPBST). After 1 h incubation at room temperature and 3x washing, bound phage were detected using HRP conjugated anti-M13 antibody (GE 27-9421-01, 1: 40,000) for 45 min at room temperature followed by 3x washing. The detection was performed using TMB substrate. The colorimetric reaction was stopped by addition of 100 μL 0.5 M H_2_SO_4_ and measured with an ELISA reader (TECAN Sunrise, 450 nm, reference 620 nm).

#### Epitope Mapping by Peptide Array

For epitope mapping by peptide array, custom microarray slides were generated by PEPperPRINT (Heidelberg). Each slide contained three copies of the TcdB array [TcdB 15mer peptides with 13 amino acids (aa) overlap (2 aa offset)]. To prepare the microarray slides, the array areas were hydrated for 15 min with 500 μL PBST [PBS 0.05% (v/v) Tween20] at room temperature and slight orbital shaking (200 rpm). To avoid unspecific binding of the scFv-Fcs, the arrays were blocked with blocking buffer (MB-070, Rockland, Limerick, USA) for 30 min (room temperature, 200 rpm agitation). After washing with PBST, 1 μg/mL (or 100 μg/mL) scFv-Fc diluted in assay buffer [PBS 0.05% (v/v) Tween20 with 10% (v/v) Rockland blocking buffer] was incubated on the array area (overnight, 4°C, 200 rpm). To remove unbound scFv-Fc, the arrays were washed three times with PBST. The scFv-Fc was detected using a DyLight 680 conjugated anti-human Fc antibody (Biomol), diluted 1:2,000 in assay buffer (30 min, room temperature, 200 rpm agitation). The array was washed three times with PBST, dipped in 1 mM Tris HCl pH 7.4 and dried in a jet of air. Slides were scanned and fluorescence signals were detected at 700 nm with an Odyssey Scanner (LI-COR Biotechnology GmbH). Afterwards, the HA-tag control peptides were stained, using anti-HA Peptide Ready Tag Mouse IgG2b (BioXcell) in a 1:5,000 dilution in assay buffer as a primary, and Anti-Mouse IgG (H+L) DyLight 680 conjugated (Cell Signaling Technology) 1:5,000 in assay buffer as a secondary antibody. Incubation with both antibodies was 30 min at RT and 200 rpm, followed by three times washing with PBST. After staining was completed, arrays were dipped in 1 mM Tris HCl pH 7.4, dried in a jet of air and used for a second scan. Analysis of the scans was performed using PepSlide Analyzer software (SICASYS Software GmbH).

## Results

### Antibody Generation

To generate antibodies against the various domains of *C. difficile* Toxin B (TcdB) a phage display approach was used. Phage display was performed using the two naïve human scFv-libraries HAL9 and HAL10 (Kügler et al., 2015). To gain a broader antibody diversity and to cover a broader range of epitopes six pannings were performed on either domains or fragments of TcdB or the full length toxin (TcdB_FL_) and at room temperature or 37°C. For two pannings, a negative preselection on the N-terminal fraction of TcdB was performed to direct the selection pressure toward antibodies that bind within the TLD, more exactly, between aa 1,128 and 1,852. An overview over the panning strategies and the success of the pannings is given in Table 1. After three rounds of panning a total of 562 clones were screened for production of TcdB specific scFv in an antigen ELISA (data not shown). On basis of the signal intensity and the signal to noise ratio in the screening ELISA, 96 scFv clones were further analyzed. Sequencing with subsequent V-gene analysis and CDR comparison using the VBASE2 tool (Mollova et al., 2010), revealed the isolation of 36 unique scFvs (Table 2). Of the 36 antibodies 15 (41%) are IGHV1, 18 (50%) IGHV3, two (5.5%) IGHV5 and one IGHV6. No IGHV2 or IGHV4 antibodies were isolated. The majority (29) of the isolated antibodies contained a lambda light chain (9x IGLV1, 10x IGLV2, 8x IGLV3, 2x IGLV6) and only 7 antibodies (18.9%) a kappa light chain (2x IGKV1, 5x IGKV3).

**Table 1:** Panning strategies.

**Panning**	**TcdB Fragment**	**°C**	**Antigen for negative selection**	**Clones screened**	**Clones analyzed**	**Unique**	**Produced and characterized as Fc-Fusion**
ViF087	TcdB_1−1852_	RT	TcdB_1−1128_	94	19	11	10
ViF088	TcdB_1−1852_	RT	no	94	13	3	3
ViF090	TcdB_1−1852_	37	TcdB_1−1128_	94	17	2	2
ViF091	TcdB_1−1852_	37	no	94	11	1	1
ViF137	TcdB_CROPs_	RT	no	94	24	12	8
SH1429	TcdB_FL_	RT	no	92	12	7	7
Σ human antibodies				562	96	36	31

*Overview over panning strategies and panning outcome*.

**Table 2:** Overview over gene families of generated antibodies.

**#**	**mAb name**	**V VH**	**D VH**	**J VH**	**V VL**	**J VL**
1	ViF087_A10	IGHV1-18*01	IGHD3-3*01	IGHJ4*02	IGLV1-44*01	IGLJ3*02
2	ViF087_B1	IGHV3-30*04	IGHD3-10*01inv	IGHJ6*02	IGLV1-47*01	IGLJ3*02
3	ViF087_B10	IGHV1-46*03	not found	IGHJ6*02	IGKV3-20*01	IGKJ4*01
4	ViF087_E1	IGHV1-69*01	IGHD2-2*01	IGHJ4*02	IGKV3-15*01	IGKJ1*01
5	ViF087_E7	IGHV1-18*01	IGHD1-26*01	IGHJ3*02	IGKV1-12*02	IGKJ4*01
6	ViF087_F1	IGHV3-33*01	IGHD4-17*01	IGHJ2*01	IGLV3-19*01	IGLJ3*01
7	ViF087_F3	IGHV1-3*01	IGHD5-12*01	IGHJ5*02	IGLV2-18*02	IGLJ3*02
8	ViF087_G10	IGHV1-69*01	IGHD3-22*01	IGHJ5*02	IGLV1-47*01	IGLJ3*02
9	ViF087_G11	IGHV3-30*01	IGHD4-17*01	IGHJ4*02	IGLV3-19*01	IGLJ3*01
10	ViF087_H5	IGHV1-46*03	IGHD4-17*01	IGHJ4*02	IGLV2-14*01	IGLJ3*02
11	ViF088_C5	IGHV3-13*01	IGHD3-16*01	IGHJ4*02	IGLV6-57*01	IGLJ3*01
12	ViF088_E10	IGHV3-30*18	IGHD6-13*01inv	IGHJ6*03	IGLV3-19*01	IGLJ3*02
13	ViF088_H10	IGHV3-33*01	IGHD3-10*01	IGHJ3*02	IGLV1-51*01	IGLJ3*02
14	ViF090_A6	IGHV1-69*01	IGHD2-15*01	IGHJ4*02	IGLV6-57*01	IGLJ3*02
15	ViF090_G5	IGHV1-3*01	IGHD5-12*01	IGHJ5*02	IGLV2-14*01	IGLJ3*01
16	ViF091_B10	IGHV1-69*06	IGHD2-21*01	IGHJ5*02	IGLV1-44*01	IGLJ3*02
17	ViF137_A3	IGHV3-23*04	IGHD6-19*01	IGHJ3*02	IGKV3-20*01	IGKJ4*01
18	ViF137_A6	IGHV3-7*01	IGHD6-13*01	IGHJ4*02	IGLV3-21*02	IGLJ3*01
19	ViF137_A9	IGHV5-51*01	IGHD1-14*01	IGHJ3*02	IGLV2-8*01	IGLJ3*02
20	ViF137_C1	IGHV3-23*04	IGHD5-5*01	IGHJ6*02	IGLV2-8*01	IGLJ3*01
21	ViF137_C2	IGHV3-23*01	IGHD5-18*01	IGHJ4*02	IGLV1-47*01	IGLJ3*02
22	ViF137_C3	IGHV1-69*01	IGHD6-13*01	IGHJ5*02	IGLV2-8*01	IGLJ3*01
23	ViF137_E4	IGHV3-21*01	IGHD6-19*01	IGHJ6*02	IGLV2-11*01	IGLJ3*02
24	ViF137_E7	IGHV5-51*01	IGHD1-1*01	IGHJ3*02	IGLV2-14*01	IGLJ3*02
25	SH1429_B1	IGHV1-3*01	IGHD5-12*01	IGHJ5*02	IGLV2-14*04	IGLJ1*01
26	SH1429_B10	IGHV3	IGHD2-8*02inv	IGHJ3*02	IGKV3-20*01	IGKJ2*01
27	SH1429_C10	IGHV3	IGHD2-15*01	IGHJ6*02	IGLV2-8*01	IGLJ3*02
28	SH1429_D6	IGHV6	IGHD2-2*03inv	IGHJ3*02	IGLV3-19*01	IGLJ1*01
29	SH1429_G1	IGHV3	IGHD2-2*02inv	IGHJ3*02	IGKV3-20*01	IGKJ4*01
30	SH1429_G6	IGHV1-18*01	IGHD3-3*01	IGHJ4*02	IGLV1-40*01	IGLJ3*02
31	SH1429_H7	IGHV3	IGHD6-19*01	IGHJ4*02	IGLV1-47*01	IGLJ3*01
32	ViF087_C12	IGHV3-48*03	IGHD3-3*01	IGHJ3*02	IGLV3-19*01	IGLJ3*02
33	ViF137_A1	IGHV1-2*02	IGHD4-17*01	IGHJ5*02	IGLV1-51*01	IGLJ3*01
34	ViF137_A5	IGHV3-30*04	IGHD5-12*01	IGHJ4*02	IGLV3-19*01	IGLJ3*01
35	ViF137_D1	IGHV1-2*02	IGHD6-13*01	IGHJ4*02	IGKV1-5*01	IGKJ1*01
36	ViF137_D4	IGHV3-23*04	IGHD4-23*01	IGHJ4*02	IGLV3-19*01	IGLJ3*01

*Antibodies not further analyzed are marked in gray*.

The scFv genes were cloned into the mammalian expression vector pCSE2.6-hIgG-Fc, which enables antibody production in HEK293-6E cells in an scFv-Fc format. After protein A purification of the scFv-Fcs from the culture supernatant, purity of the mAb preparations was controlled by SDS-PAGE and Coomassie staining (data not shown). There were no visible impurities or breakdown products. Of the 36 mAbs, 31 were successfully produced and further analyzed.

### Validation of Antigen Binding and Domain Mapping

**Figure 2:**
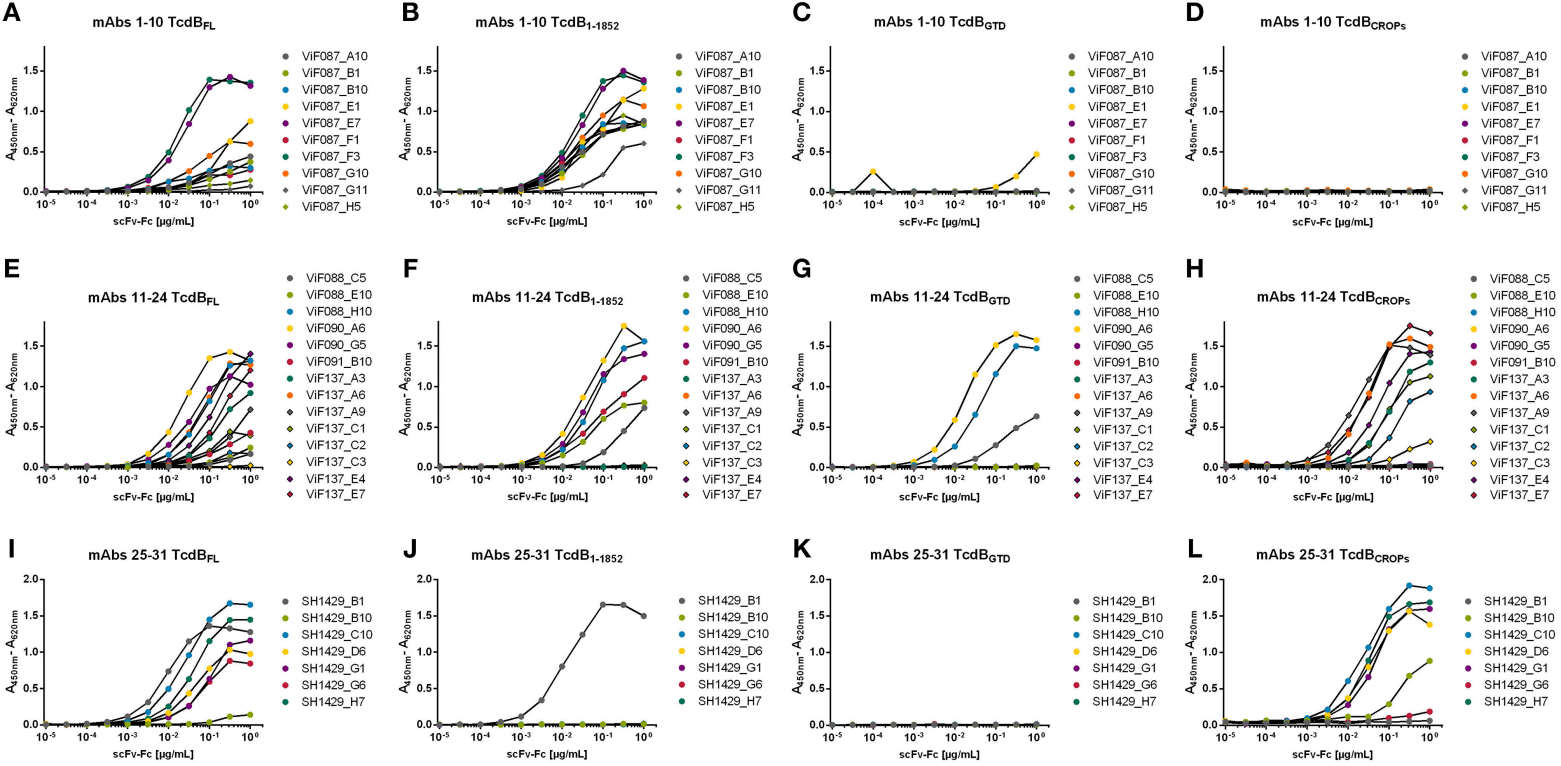
Antigen ELISA on TcdB variants. 31 mAbs were tested for binding on 100 ng of immobilized fragments of TcdB or full length TcdB. Bound scFv-Fcs were detected using an HRP conjugated anti-human Fcγ antibody. **(A,E,I)** Titration on TcdB_FL_; **(B,F,J)** Titration on TcdB_1−1852_; **(C,G,K)** TcdB_GTD_ was used as antigen; **(D,H,L)** Titration on TcdB_CROPs_.

The 31 scFv-Fcs were analyzed by titration ELISA on four different TcdB variants (TcdB_FL_, TcdB_1−1852_, TcdB_GTD_ and TcdB_CROPs_) first, to verify that format change from scFv to the bivalent scFv-Fc did not impair antigen recognition, second, to analyse TcdB_FL_ binding of antibodies that were generated on TcdB fragments, third, to validate antibody TcdB fragment specificity and fourth, to determine the binding domains or regions of the respective mAbs (Figure 2 and Table 3).

**Table 3:** Overview over binding characteristics of all 31 antibodies analyzed in this study, including Antigen ELISA, Immunoblot and epitopes.

**#**	**mAb name**	**Antigen (Panning)**	**TcdB variants bound in ELISA**	**WB**	**Epitope region (Peptide array; position of 1^st^ aa of 15mer peptide)**	**Minimal epitope region (MER, phage display)**	**No of clones contributing to MER**
1	ViF087_A10	TcdB_1−1852_	TcdB_FL_, (≤ 50%), TcdB_1−1852_	–	aa 402–404 and 412–422	aa 423–433	5
2	ViF087_B1	TcdB_1−1852_	TcdB_FL_, (≤ 50%), TcdB_1−1852_	+/–		No hits	–
3	ViF087_B10	TcdB_1−1852_	TcdB_FL_, (≤ 50%), TcdB_1−1852_	+/–	aa 288–294 (not exclusively)	aa 289–313	1 (one hit in ELISA)
4	ViF087_E1	TcdB_1−1852_	TcdB_FL_, TcdB_1−1852_, TcdB_GTD_	+/–	aa 522	aa 528–543	16
5	ViF087_E7	TcdB_1−1852_	TcdB_FL_, TcdB_1−1852,_	+/–		No hits	–
6	ViF087_F1	TcdB_1−1852_	TcdB_FL_, (≤ 50%), TcdB_1−1852,_	–		No hits	–
7	ViF087_F3	TcdB_1−1852_	TcdB_FL_, TcdB_1−1852,_	+/–		No hits	–
8	ViF087_G10	TcdB_1−1852_	TcdB_FL_, TcdB_1−1852,_	–		No hits	–
9	ViF087_G11	TcdB_1−1852_	TcdB_FL_, TcdB_1−1852,_	–		No hits	–
10	ViF087_H5	TcdB_1−1852_	TcdB_FL_, TcdB_1−1852,_	+/–		No hits	–
11	ViF088_C5	TcdB_1−1852_	TcdB_FL_, TcdB_1−1852_, TcdB_GTD_	+/–		No hits	-
12	ViF088_E10	TcdB_1−1852_	TcdB_FL_, TcdB_1−1852,_	–		No hits	–
13	ViF088_H10	TcdB_1−1852_	TcdB_FL_, TcdB_1−1852_, TcdB_GTD_	+		aa 24–84	12
14	ViF090_A6	TcdB_1−1852_	TcdB_FL_, TcdB_1−1852_, TcdB_GTD_	+		aa 23–83	4
15	ViF090_G5	TcdB_1−1852_	TcdB_FL_, TcdB_1−1852,_	–		No hits	–
16	ViF091_B10	TcdB_1−1852_	TcdB_FL_, (≤ 50%), TcdB_1−1852_	–		No hits	–
17	ViF137_A3	TcdB_CROPs_	TcdB_FL_, TcdB_CROPs_	+	aa 2342–2344 (not exclusively)	aa 2284–2364	6
18	ViF137_A6	TcdB_CROPs_	TcdB_FL_, TcdB_CROPs_	+	aa 2340–2344	aa 2275–2364	23
19	ViF137_A9	TcdB_CROPs_	TcdB_FL_, (≤ 50%), TcdB_CROPs_	+	KYYF° (° = D or N) aa 1854–1862, 2080–2086, and 2212–2216	aa 1858–1868	13
20	ViF137_C1	TcdB_CROPs_	TcdB_FL_, (≤ 50%), TcdB_CROPs_	+		No hits	–
21	ViF137_C2	TcdB_CROPs_	TcdB_FL_, (–), TcdB_CROPs_	+		No hits	–
22	ViF137_C3	TcdB_CROPs_	TcdB_CROPs_ (–)	+	aa 1922–1938	aa 1860–1992	3
23	ViF137_E4	TcdB_CROPs_	TcdB_FL_, TcdB_CROPs_	+	aa 2342–2344	aa 2292–2364	18
24	ViF137_E7	TcdB_CROPs_	TcdB_FL_, TcdB_CROPs_	+	*KYYF° (* = I, S or D; ° = D or N) aa 1852–1860, 2078–2084, and 2212–2216	2 epitopes, shared motif: DKYYFNP aa 1862–1868 and 2220–2226	17 + 5
25	SH1429_B1	TcdB_FL_	TcdB_FL_, TcdB_1−1852_	+/–	aa 414–424	aa 423–432	8
26	SH1429_B10	TcdB_FL_	TcdB_FL_, TcdB_CROPs_	–		aa 2267–2364	19
27	SH1429_C10	TcdB_FL_	TcdB_FL_, TcdB_CROPs_	+	aa 2340–2342	aa 2333–2363	15
28	SH1429_D6	TcdB_FL_	TcdB_FL_, TcdB_CROPs_	+	aa 2078 and 2084	aa 2010–2118	6
29	SH1429_G1	TcdB_FL_	TcdB_FL_, TcdB_CROPs_	+	aa 2316–2324	aa 2267–2364	10
30	SH1429_G6	TcdB_FL_	TcdB_FL_, TcdB_CROPs_ (–)	+		aa 2228–2291	4
31	SH1429_H7	TcdB_FL_	TcdB_FL_, TcdB_CROPs_	+		aa 2276–2364	11

*Result of western blot shown as +: strong binding to linearized TcdB or its fragments, +/–: mixed results, –: no binding to linearized TcdB or its fragments*.

In this ELISA setup, all mAbs bound to their respective panning TcdB variant in a concentration dependent manner. The antibodiesViF137_C3 (Figure 2H) and SH1429_B10 (Figure 2I) showed only weak binding in ELISA.

Interestingly, almost no binding to TcdB_FL_ was detected for some mAbs [ViF087_G11, ViF087_H5 (Figure 2A), ViF088_C5, ViF088_E10, and ViF137_C2 (Figure 2E)] despite of binding to the panning antigen. For further seven antibodies [ViF087_A10, ViF087_B1, ViF087_B10, ViF087_F1 (Figure 2A), ViF091_B10, ViF137A9, and ViF137_C1 (Figure 2E)] the binding to TcdB_FL_ was notably reduced (≤ 50% signal intensity at highest concentration) compared to the respective panning TcdB variant.

As expected, there was no binding to TcdB_1−1852_ or TcdB_GTD_ of mAbs that were generated by panning against TcdB_CROPs_ [mAbs ViF137 (Figures 2F,G)] and no binding to TcdB_CROPs_ of mAbs that were generated by panning against TcdB_1−1852_ [mAbs ViF087, ViF088, ViF090, and ViF091_B10 (Figures 2D,H)], proving domain specificity of the mAbs generated in this study.

Of the 31 mAbs tested in this assay, 14 bound to TcdB_CROPs_, among them all antibodies derived from the panning against TcdB_CROPs_ [mAbs ViF137 (Figure 2H)], as well as the majority of the antibodies derived from the panning against TcdB_FL_ [SH1429_B10, SH1429_C10, SH1429_D6, SH1429_G1, SH1429_G6 (only very weak), and SH1429_H7 (Figure 2L)].

Four mAbs [ViF087_E1, ViF088_C5, ViF088_H10, and ViF090_A6 (Figures 2C,G)] bound to TcdB_GTD_ indicating an epitope within the GTD domain of TcdB. The remaining 13 antibodies bound to immobilized TcdB_1−1852_, but not to TcdB_GTD_. Therefore, it is likely that these antibodies bound to epitopes between aa 543 and 1,852, but due to possible differences in protein folding between the different TcdB variants, a lack of TcdB_GTD_ binding in this experiment is not sufficient to exclude an epitope within this domain.

### *In vitro* Neutralization

In a next step, the antibodies were tested for *in vitro* neutralization of TcdB. Therefore, a cell based assay with Vero cells (African green monkey kidney cells) was chosen. Upon cellular uptake of TcdB and release of the GTD into the cytosol, the GTD glucosylates small Rho GTPases which impairs several cell signaling pathways and consequently leads to a breakdown of the actin cytoskeleton. This effect induces morphological changes like the formation of retraction fibers and finally cell rounding (Just et al., 1995). Neutralization efficacy of an antibody was analyzed by comparing the percentage of round cells in samples of cells treated with TcdB to cells treated with TcdB which was preincubated with an antibody.

To determine the optimal working concentration of TcdB_FL_ for this assay, a serial dilution of TcdB in culture media was applied to Vero cells. After 5 h, pictures of the cells were collected and the percentage of round cells was determined by counting using ImageJ software. Cell rounding correlated with TcdB_FL_ concentration (see Supplementary Figure 1). For following neutralization experiments, a TcdB_FL_ concentration of 0.1 pM (resulting in around 80% cell rounding) was coincubated with 100 nM mAb in cultivation media and subsequently transferred to the Vero cells.

To determine neutralization efficacy, the percentage of round cells was determined and normalized to the percentage of round cells in the control wells (Vero cells w/o TcdB_FL_ and Vero cells with TcdB_FL_ w/o mAb).

**Figure 3:**
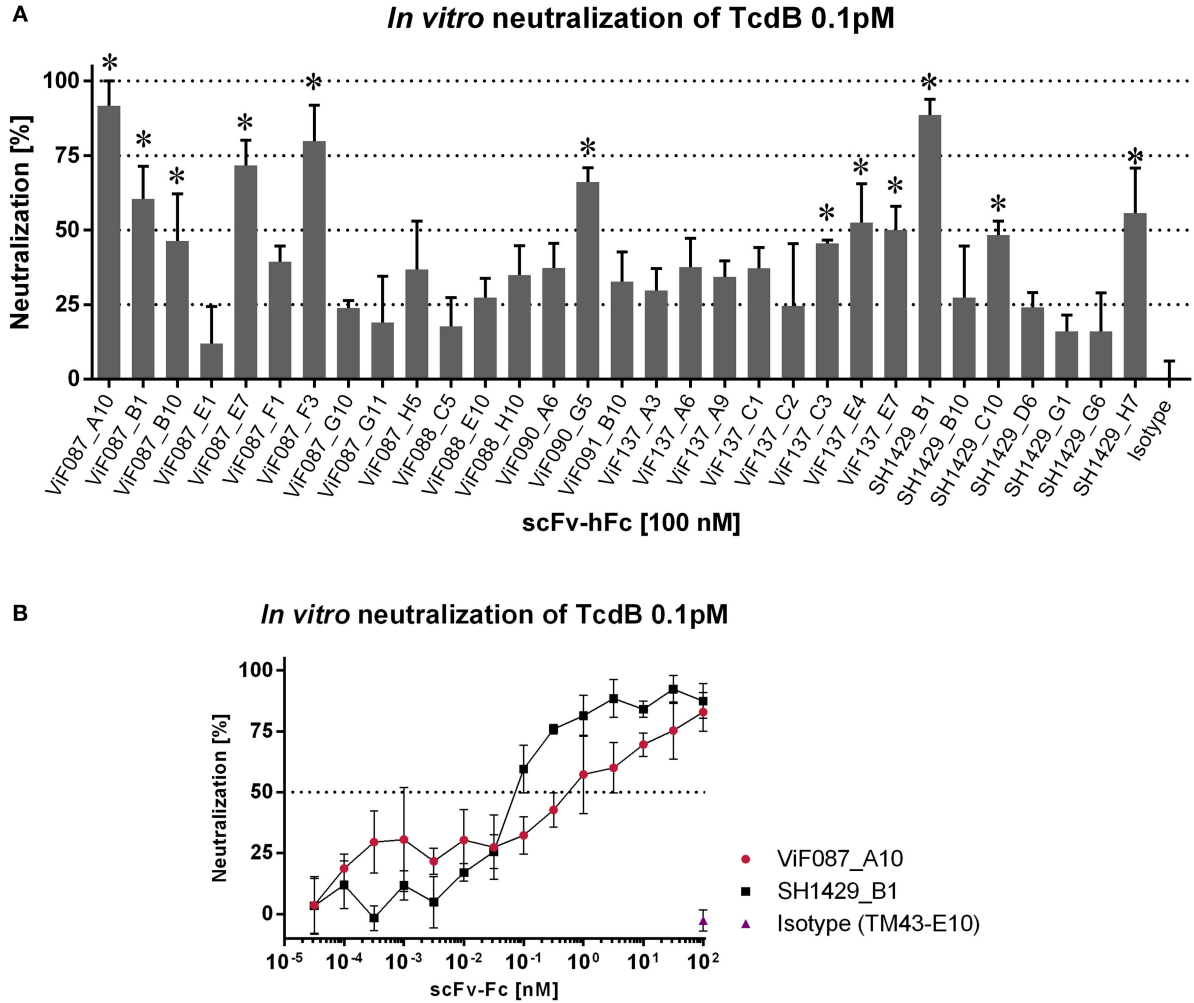
*In vitro* neutralization of TcdB. TcdB (0.1 pM) and mAbs were coincubated in cultivation media and transferred to subconfluent Vero cells in 96 well plates. Pictures of each well were taken when cell rounding in NK control wells (TcdB w/o antibody) was 70–80%. Number of round cells were normalized to NK control and percentage of round cells after media exchange (w/o toxin or antibody) was set to 100% neutralization. **(A)** Initial screening for neutralization using all 31 mAbs in a 10,000-fold molar excess. Bars represent technical triplicates with SD as error bars. A one-way ANOVA test was performed for each antibody against the isotype control TM43_E10 (Kügler et al., 2015) *p < 0.0001. **(B)** IC_50_ of ViF087_A10 and SH1429_B1 were estimated with serial antibody dilutions.

In a first screening for neutralization, all tested mAbs reduced the percentage of round cells after TcdB treatment to some extent, with significant neutralization (*p* > 0.0001) seen for 12 of 31 mAbs (Figure 3A). Preincubation of TcdB with either ViF087_B1, ViF087_E7, ViF090_G5, ViF137_E4 or SH1429_H7 resulted in more than 50% neutralization. Best *in vitro* neutralization of TcdB was achieved with ViF087_A10, ViF087_F3, or SH1429_B1 (>75% reduction of cell rounding).

TcdB_FL_ neutralization of ViF087_A10 and SH1429_B1 was further verified using serial dilutions of these antibodies (Figure 3B). ViF087_F3 was not included since it contains the same heavy chain as SH1429_B1 which suggests a neutralization via the same mechanism, and size exclusion chromatography revealed partial aggregation of ViF087_F3 which was not seen for SH1429_B1 (data not shown). The dilution series confirmed the results of the neutralization screening. At the starting concentration of 100 nM ViF087_A10 or SH1429_B1 nearly completely inhibited the cell rounding induced by TcdB_FL_ intoxication (Figure 3B). However, the dilution series revealed that SH1429_B1 is roughly 10 times more potent than ViF087_A10, since 0.1 nM SH1429_B1 was sufficient to reduce cell rounding by ~50% whereas 1 nM of ViF087_A10 was needed to achieve a comparable effect.

Next, a combination of ViF087_A10 and SH1429_B1 was tested to neutralize TcdB_FL_ in an *in vitro* assay, but neutralization achieved with this combination was not stronger than for SH1429_B1 alone (data not shown).

Unlike Bezlotoxumab, a human anti-TcdB antibody already approved by the FDA for therapy of recurrent CDI (Navalkele and Chopra, 2018), the two most potent neutralizing antibodies generated in this study (ViF087_A10 and SH1429_B1) bind to epitopes within TcdB_1−1852_ (Figures 2B,J). Bezlotoxumab binds to two epitopes within the CROPs. There are two distinct neutralization mechanism possible for Bezlotoxumab: i) blocking of interaction with carbohydrate structures and therefore inhibition of cell binding (Orth et al., 2014), ii) inhibition of binding to CSPG4 (Gupta et al., 2017). However, this antibody proves that neutralization via this repetitive domain is feasible. Nevertheless, there are three cellular receptors for TcdB described that bind to specific receptor binding sites within the TLD, e.g., members of the frizzled family binding to TcdB_1285−1804_ with low nanomolar affinity (LaFrance et al., 2015; Yuan et al., 2015; Tao et al., 2016; Gupta et al., 2017; Chen et al., 2018). Hence it might be possible that the anti-TcdB_CROPs_ antibodies generated in this study reduce CROPs mediated binding of TcdB to the cell surface but that TcdB induced cell rounding is not reduced because of the numerous compensation mechanisms mediated by the additional cell surface receptors. In this case a combination of the mAbs directed against TcdB_CROPs_ with mAbs that bind to the N-terminal domains could lead to improved neutralization and synergistic effects. To test this hypothesis, combinations of either ViF087_A10 or SH1429_B1 with a mAb directed against TcdB_CROPs_ were tested in an *in vitro* TcdB neutralization assay. Therefore, 0.1 pM of TcdB was preincubated with 1 nM ViF087_A10 or 0.1 nM SH1429_B1 [the concentration needed for ~50% TcdB neutralization as determined by titration (Figure 3B)] and 100 nM of either of the 14 anti-TcdB_CROPs_ mAbs for 30 min at room temperature and then transferred to Vero cells. TcdB neutralization for each antibody combination was compared to neutralization achieved with ViF087_A10, or SH1429_B1 alone in the same assay.

**Figure 4:**
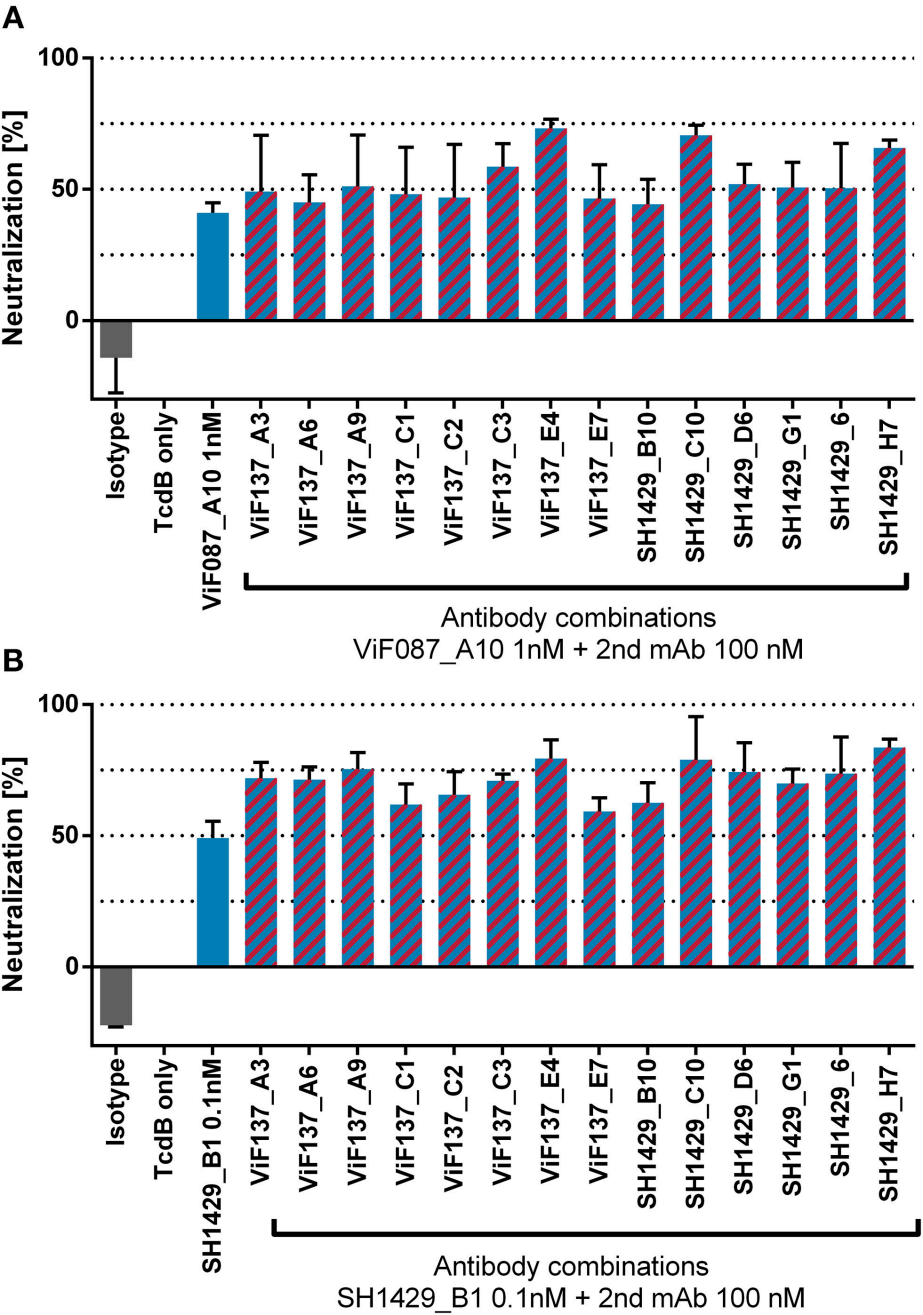
*In vitro* neutralization of TcdB with antibody combinations. TcdB (0.1 pM) and either 1 nM of ViF087_A10 **(A)** or 0.1 nM SH1429_B1 **(B)** mAbs were coincubated in cultivation media with 100 nM of CROPs binding mAbs.

Forty-one percent neutralization was observed for 1 nM ViF087_A10 in this assay. Addition of 100 nM of a second mAb directed against TcdB_CROPs_ improved neutralization. The best neutralization of ViF087_A10 was achieved in combination with ViF137_E4 (73%), SH1429_C10 (70%) and SH1429_H7 (66%) (Figure 4A).

0.1 nM of mAb SH1429_B1 reduced cell rounding by 49%. As for ViF087_A10, addition of 100 nM of an anti-TcdB_CROPs_ mAb increased neutralization and best neutralizing with SH1429_B1 was achieved in combination with the same TcdB_CROPs_ binders as for ViF087_A10, namely ViF137_E4 (79%), SH1429_C10 (78.9%) or SH1429_H7 (83.6%) (Figure 4B). Remarkably, the anti-TcdB_CROPs_ mAbs leading to the highest increase of neutralization with either ViF087_A10 or SH1429_B1 in this assay were the same mAbs that already showed best neutralization among TcdB_CROPs_ binders in the initial screening as single antibodies (ViF137_E4 53%, SH1429_C1048%, SH1429_H7 56%). Therefore, the increase of neutralization in this combinatory assay seems to be based on additive rather than synergistic effects.

### Epitope Mapping

**Figure 5:**
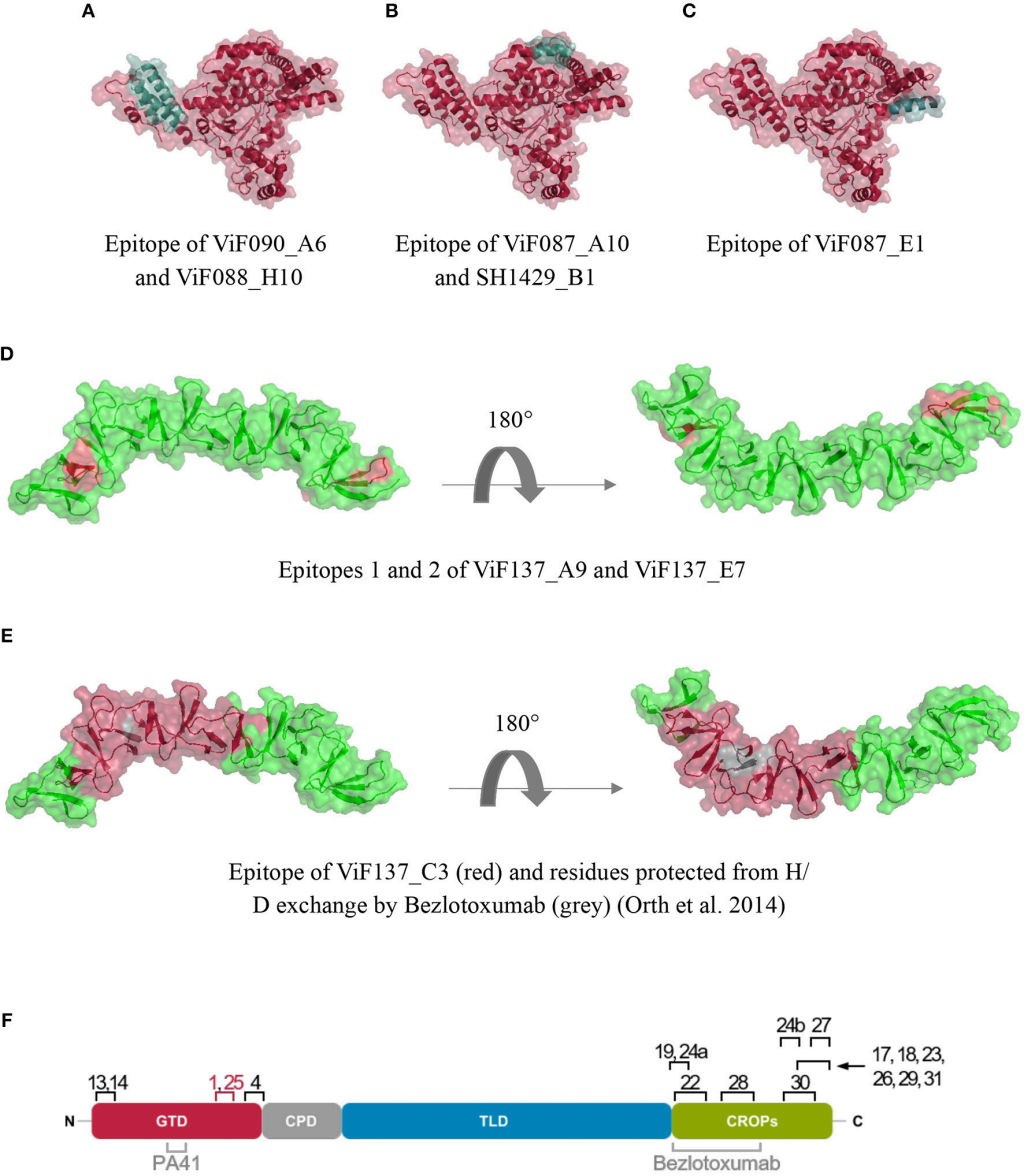
Crystal structures of the glucosyltransferase of TcdB (**A–C**, PDB 2bvm), the N-terminal (**D,E** PDB 4np4) and C-terminal (**F**, PDB 4nc2) fraction of the CROP domain with highlighted epitopes of mapped antibodies. Figures were created using PyMOL (De Lano, 2002) **(F)** overview over epitopes mapped to TcdB in this study. Neutralizing epitopes are highlighted in red. Neutralizing epitopes published by others are designated in gray (Orth et al., 2014; Kroh et al., 2018).

To identify the epitopes of ViF087_A10 and SH1429_B1 that elicit neutralization, and gain more information about the binding sites of the other antibodies, epitope mapping was performed. To generate reliable data and to increase the chance of identifying the epitopes or at least to narrow down the binding regions of a significant number of antibodies, two different approaches were used. First, all antibodies were tested on a peptide array consisting of 15mer peptides of TcdB. The maximum resolution of this array was two amino acids due to the 2 aa offset of two neighboring peptides on the array. This approach was successful for about 40% of the antibodies (Table 3). Two antibodies (ViF137_A9 and ViF137_E7) were found to exclusively bind to three clusters of peptides within the CROP domain (1858–1869, 2084–2095, 2216–2227, and 1858–1867, 2084–2093, 2218–2225, respectively) (Figure 5D). These clusters contain a common motif which probably resembles the key amino acids necessary for antibody-antigen interaction [KYYF^†^ (^†^ = D or N) and ^*^KYYF^†^ (^*^ = I, S or D, ^†^ = D or N), respectively]. The two neutralizing antibodies ViF087_A10 and SH1429_B1 both specifically bound to the five 15mer peptides starting between aa 414 and 422. ViF087_A10 additionally also bound to the peptides starting at aa position 402 and 404 (Supplementary Figure 2).

For other antibodies, the results of the peptide arrays were less clear.

For mAb ViF087-B10, a region within the GTD, represented by four neighboring peptides starting at aa positions 288, 290, 292, and 294 was highly overrepresented among the fifteen peptides showing the highest signal in the peptide array. Nevertheless, this antibody seemed to have a broader cross-specificity as the remaining signals originated from different regions of the toxin and did not have common features. MAb ViF087-E1 strongly, but not exclusively, bound to a peptide that spans aa 522–537.

Furthermore, antibodies that bind to epitopes within the CROPs, namely ViF137_A3, ViF137_A6, ViF137_C3, SH1429_C10, SH1429_D6, and SH1429_G1, reacted with peptides derived from the CROPs, but they did not exclusively bind to only one cluster of neighboring peptides, but showed a higher degree of cross-specificity or tolerance of aa substitutions, therefore epitope identification only by peptide array was not feasible for these antibodies. As expected, especially for short (i.e., *linear*) epitopes, the peptide array approach yielded good results.

Thus, to confirm the epitopes found by peptide array and to identify further epitopes or at least binding regions of the remaining antibodies, a phage display approach was used. Therefore, a *tcdB*-gene fragment phage display library was generated and packaged with Hyperphage (M13K07ΔgIII) to improve the presentation of correct open reading frames (Supplementary Table 1). This library was used for pannings on immobilized mAbs and/or for panning in solution with subsequent immunoprecipitation using protein A coupled magnetic beads. *E. coli* clones, carrying *tcdB*-gene fragments coding for the potential epitope region, were identified by monoclonal phage ELISA on immobilized mAbs. For 17 out of 31 mAbs the screening ELISA resulted in the identification of monoclonal phage presenting TcdB fragments that specifically bound to the corresponding anti-TcdB-mAb but not to an irrelevant control mAb (data not shown). The related gene fragments were sequenced, translated into aa sequences and aligned to the TcdB sequence. For each mAb, the stretch of amino acids that was covered by all sequenced clones that carried correct inserts in the pHORF3 vector, is referred to as the minimal epitope region (MER) (Table 3).

For two antibodies from pannings against TcdB_1−1852_, ViF088_H10, and ViF090_A6, an almost identical MER in the N-terminal part of the GTD was identified, comprising of aa 24–84 and 23–83, respectively. These aa make up three alpha helices that belong to an alpha helical bundle at the toxins N-terminus (Figure 5A).

In the screening ELISA for ViF087_B10 only one clone tested showed specific binding to its mAbs. This clone carried a TcdB fragment spanning aa 289–313 which is in line with the result from the peptide array.

The MER of the two best neutralizing antibodies generated in this study (ViF087_A10 and SH1429_B1) is also located in the GTD and comprises aa 423–433. For ViF087_A10, the signal-to- noise ratio of epitope presenting clones in screening ELISA was comparably low, nevertheless the MER was in accordance to the binding pattern on the peptide array for both antibodies. As shown in Figure 5B, the MER is located directly next to the Rho-GTPase binding groove of TcdB.

The MER of ViF087_E1 was located between aa 528 and 543, which corresponds to the C-terminus of the GTD (Figure 5C) The MER overlaps with the peptide (aa 522–537) found with the peptide array. For ViF137_A9 and ViF137_E7 the MERs identified by phage display were in accordance with the results of the peptide array, albeit for ViF137_A9 only one and for ViF137_E7 only two of the three clusters were confirmed (Figure 5D).

The MER of ViF137_C3 was located in the N-terminal portion of the CROPs and spans aa 1860–1992. For SH1429_D6, the MER spanned a stretch of 118 amino acids in the middle of the CROPs (aa 2010–2118) and the MER of SH1429_G6 aa 2228–2291.

The seven remaining mAbs (ViF137_A3, ViF137_A6, ViF137_E4, SH1429_B10, SH1429_C10, SH1429_G1, and SH1429_H7) for which the phage display approach was successful, all bind to the C-terminal part of TcdB, therefore the MERs resemble the last 70–130 aa of the toxin's C-terminus.

For ViF137_A3, ViF137_A6, ViF137_C3, (Figure 5E), SH1429_C10, SH1429_D6, and SH1429_G1 a re-evaluation of the results of the peptide array was done after identification of the MERs by phage display. In all cases peptide clusters were identified that interacted with the respective antibodies and which were in accordance to the MERs identified by phage display (Table 3).

## Discussion

The toxins TcdA and TcdB are main virulence factors for CDI. The anti-TcdB antibody Bezlotoxumab was approved in 2016 by the FDA for prevention of CDI recurrence. The corresponding antibody Actoxumab, directed against TcdA, did not show clinical efficacy in clinical phase 3^1^. In the clinical phase three study (MODIFY II) the CDI recurrence was reduced from 26 to 16% by Bezlotoxumab (Wilcox et al., 2017). Because of this limited efficacy and the fact that currently three different TcdB receptors and a potential carbohydrate structure (Greco et al., 2006; LaFrance et al., 2015; Yuan et al., 2015; Tao et al., 2016; Gupta et al., 2017) are described as interaction partners, new studies to describe neutralizing and non-neutralizing epitopes are necessary, for further development of antibody combinations that potentially improve clinical outcome. For these reasons, we generated a set of novel human monoclonal antibodies targeting TcdB using phage display.

We performed a total of six antibody selections using different fragments/ functional domains of TcdB, and different panning strategies. The panning strategies differed in regards of the TcdB fragments and temperature used. Since all six panning strategies led to the identification of unique and specific antibodies, enrichment of specific antibody phage directed against the different TcdB variants was successful in all cases. Sequencing revealed the isolation of a total of 36 unique human antibodies. In accordance to previous studies, pooling of lambda and kappa libraries led to an enhanced but not exclusive selection of lambda antibodies (80%) and also the subfamily distribution of selected antibodies represents the pattern that was described before (Kügler et al., 2015).

For further validation and characterization, the antibody candidates were converted into the IgG like bivalent scFv-Fc format. Due to a fast cloning and better production rates, this format is preferred over the full IgG format for the rapid screening of a higher number of candidate antibodies (Bujak et al., 2014; Rasetti-Escargueil et al., 2017).

Thirty-one antibodies were tested for antigen binding and domain specificity in an antigen ELISA on four different TcdB variants (TcdB_FL_, TcdB_1−1852_, TcdB_CROPs_, and TcdB_GTD_). By antigen ELISA on the respective panning antigen, we validated that the antibody antigen interaction was not impaired by the format change from scFv to scFv-Fc and switch of the production system, since all antibodies bound to their respective antigen, albeit 2 out of 31 weaker.

Interestingly, for five antibodies almost no binding to TcdB_FL_ was detected and for further seven antibodies the binding to TcdB_FL_ was drastically reduced compared to the respective panning antigen. All these antibodies were generated on fragments of TcdB, therefore the epitopes or binding regions of these antibodies might not be accessible in the tertiary structure of the full length TcdB or not folded correctly in the fragments. For TcdA a 3D model of the holotoxin on the basis of electron micrographs reveals the domain organization of the toxin (Pruitt et al., 2010). In this model the CROPs form a long tail that lays back onto the N-terminal portion of the toxin. Electron micrographs of TcdB suggest a similar domain organization in this homologous toxin (Pruitt et al., 2010). Epitope regions that are located at the interface between CROPs and the N-terminal portion of the toxin may be less accessible in the full length toxin due to steric hindrance, which could explain reduced antibody binding on full length TcdB. Different antibody binding on TcdB fragments and full length TcdB was also shown by Chung and coworkers (this issue of Frontiers Microbiology).

Fourteen of the antibodies characterized in this study bind to TcdB_CROPs_, 14 to TcdB_1−1852_ but not to TcdB_GTD_. Despite of depletion of the library by incubation on immobilized TcdB_1−1128_ prior to the panning in cases of panning ViF087 and ViF090, four antibodies were obtained that bind to TcdB_1−1852_ and TcdB_GTD_. For the domain mapping ELISA, an enzymatically inactive GTD mutant (D286/288N) was used. Even though there are only two amino acids exchanged, the overall structure of the domain might be changed. Therefore, epitopes might not be accessible or conformational epitopes might be destroyed. Loss of antibody binding after mutation of single amino acids had already been described in the literature for antibodies targeting the diphtheria DT toxin (Bigio et al., 1987), among many others. To avoid the generation of antibodies which are not binding the full length protein, antibody generation strategies could be applied with alternating panning rounds on full length protein and protein fragments to focus on a particular epitope (Thie et al., 2011).

Since CROPs, GTD, and TLD of TcdB all harbor epitopes that can be targeted for neutralization (Babcock et al., 2006; Marozsan et al., 2012; Wang et al., 2012; Maynard-Smith et al., 2014; Anosova et al., 2015) all antibodies were subsequently tested in an *in vitro* TcdB neutralization assay which is based on cell rounding of Vero cells. All 31 mAbs reduced the percentage of round cells after TcdB treatment to some extent and therefore had slight neutralizing effects. However, of the 14 antibodies directed against the CROPs domain only two (ViF137_E7 and SH1429_H7) had neutralization efficacies of more than 75%. A previous study showed, that generation of neutralizing antibodies against TcdB CROPs is difficult through immunization as well (Maynard-Smith et al., 2014). Based on homology to TcdA, the CROPs of TcdB were proposed to harbor 4 putative carbohydrate binding sites (Greco et al., 2006; Orth et al., 2014) which may contribute avidity effects upon cell binding. These binding sites share structural similarity (repetitive elements) but differ in respect to their amino acid sequence, therefore it may be difficult to develop a single antibody that completely blocks all interactions between the CROPs and the carbohydrate structures. As revealed by crystal structure analysis, the already approved therapeutic antibody Bezlotoxumab binds to two epitopes within the CROPs, therefore it was suggested that this antibody blocks interaction of the CROPs with carbohydrate structures on the cell surface (Orth et al., 2014). Nevertheless, binding to aa 1878–1961 also inhibits interaction with CSPG4 receptor (Gupta et al., 2017) and due to the existence of two epitopes within the CROP domain also aggregation of the toxin as neutralization mechanism cannot be excluded. As of today, it is not clear which of the above mentioned is the major neutralization mechanism of Bezlotoxumab.

The anti-CROPs mAbs generated in this study did not lead to a substantially increased neutralization when applied together with mAbs directed against the N-terminal fraction of TcdB (aa 1–1852), showing at most an additive effect.

The two best neutralizers ViF087_A10 and SH1429_B1 with neutralizing IC_50_ values of ~1 nM and ~0.1 nM, respectively, are directed against the N-terminal fraction of TcdB (aa 1–1852). Via antigen ELISA it was not possible to narrow down the binding region to a single domain. Therefore, an epitope mapping via peptide array and phage display was performed. In these assays all antibodies were included with the hope to identify correlations between epitopes and neutralization efficacy. Unfortunately, but not unexpectedly, epitope mapping by peptide array was not successful for the majority of the tested antibodies. Since a prerequisite for binding of antibodies to peptides immobilized on the array surface is that the antibodies bind to continuous epitopes not including complex folding (Abbott et al., 2014), this result suggests that most of the identified human antibodies are very likely to bind to complex conformational and/or discontinuous epitopes. This hypothesis was also supported by data from immunoblot assays, where most antibodies did not bind to denatured, linearized TcdB (example given in Supplementary Figure 3). Nevertheless, even though ViF087_A10 does not bind to denatured TcdB in immunoblot and SH1429_B1 only very weakly, the core of their epitope is a continuous aa stretch within the GTD (aa 423–433 and 423–432, respectively). This epitope, primarily found by peptide array, was also confirmed by antigen fragment phage display, a method that also allows the identification of conformational epitopes (Cariccio et al., 2016). Remarkably, both neutralizing antibodies share the same epitope that is a surface exposed α-helical secondary structure located in close proximity to the substrate binding groove for the small Rho-GTPases (Figure 5B).

This epitope is well conserved between the clinically most relevant strains of *CDiff* clade 1 and clade 2 (hypervirulent strains), however strains of the TcdA^−^ TcdB^+^ in clade 4 show some variance in this region.

Due to the localization of the epitope, at least two neutralization mechanisms are possible for ViF087_A10 and SH1429_B1: (i) inhibition of substrate binding or (ii) sterical hindrance of TcdB_GTD_ translocation through the pore. The latter mode of action was recently described for the humanized monoclonal antibody PA41 that binds to aa 290–360 (Kroh et al., 2018).

For one antibody, ViF137_C3, an epitope (aa 1860–1992) was found that includes one of the epitopes described for Bezlotoxumab where aa 1902–1907 were shown to be partially protected from H/D exchange by bezlotoxumab (Orth et al., 2014). Nevertheless, TcdB neutralization achieved with this antibody in cell rounding assays was only < 50%. Unfortunately, the minimal epitope region identified by phage display was larger, thus not allowing conclusions on whether our antibody interacts with the same amino acids of TcdB.

For most antibodies that exclusively bind to TcdB_1−1852_ and TcdB_FL_ in antigen ELISA it was not possible to determine the epitope by neither of the methods tested. These mAbs probably bind to complex discontinuous epitopes, including aa that are located far apart on the primary structure of TcdB and only come into close proximity upon folding of the polypeptide chain. Such epitopes might be hard to display on phage due to a selection pressure toward smaller peptides during library packaging (Kügler et al., 2008) and potential misfolding of the antigen fragments.

In conclusion, a panel of novel fully human monoclonal antibodies was generated that target TcdB of *C. difficile* (Figure 5F). A new neutralizing epitope was found located within the GTD of TcdB. For future development of neutralizing antibodies, the following regions may be addressed (i) aa 290–360 (Kroh et al., 2018), (ii) aa 423 432 (epitope of the two best neutralizers generated in this study), (iii) aa 1372-1493, involved in binding to poliovirus receptor- like protein-3 (LaFrance et al., 2015; Manse and Baldwin, 2015), (iv) aa 1810–1850, involved in CSPG4 binding (Gupta et al., 2017) and aa 1430–1600 containing the binding site for FZD- cysteine rich domain (Chen et al., 2018), whereas the following regions may be omitted: (i) N-terminus and C-terminus of GTD, since antibodies against these regions generated in this study did not show significant neutralization, (ii) the C-terminus of CROPs (Maynard-Smith et al., 2014; Gupta et al., 2017).

## Author Contributions

VF, PH, SH, SG, and JH performed experiments. VF, SG, FL, SD, RG, and MH planned and analyzed experiments. SD, RG, and MH conceived the study. VF, SD, RG, and MH wrote the manuscript. All authors contributed to the final manuscript.

We are grateful to Helma Tagte, Hannover Medical school, Institute for Toxicology, for producing TcdB and the fragments thereof.

### Conflict of Interest Statement

VF, SD, FL, RG and MH are inventors on a patent application regarding the novel neutralizing epitope within the GTD and corresponding antibodies. They hereby declare that the patent application does not alter their adherence to all Frontiers in Microbiology policies on sharing data and materials. The remaining authors declare that the research was conducted in the absence of any commercial or financial relationships that could be construed as a potential conflict of interest.

## Footnotes

^1^Merck Newsroom Home (2015). Available online at: https://www.mrknewsroom.com/ (Accessed September 26, 2018).
