# Supplementary material for: Development of Neutralizing and Non-neutralizing Antibodies Targeting Known and Novel Epitopes of TcdB of Clostridioides difficile
Source: Front Microbiol. 2018 Dec 6;9:2908. doi: 10.3389/fmicb.2018.02908 (PMC6291526; doi:10.3389/fmicb.2018.02908)
Supplement: Supplementary file 1 [file Data_Sheet_1.PDF]

Supplementary Figures and Tables

Supplementary S1: Titration of the TcdB in cell rounding assay

In vitro intoxication of Vero cells using a serial dilution on $TcdB_{FL.}$

Supplementary S2: Peptide Array results (examples)

Antibody binding to TcdB-peptides on a peptide array.

Supplementary S3: Immunoblot of scFv-Fc Fragments on $TcdB_{FL}$

Immunoblot of TcdBFL under reducing conditions. 5µg TcdB were applied to a SDS Gel and detected using 1µg/mL of mAb per lane.

Table S1: Sequencing analysis of TcdB fragment library

Library size (independent clones)	1,9*10^7	
In frame insert rate after packaging	65 %	data of packaged library before panning (n=91)
Mean insert length	39 AA	
Shortest fragment found	13 AA	
Longest fragment found	73 AA	
