# Supplementary figures and images for: Development of Neutralizing and Non-neutralizing Antibodies Targeting Known and Novel Epitopes of TcdB of Clostridioides difficile
Source: Front Microbiol. 2018 Dec 6;9:2908. doi: 10.3389/fmicb.2018.02908 (PMC6291526; doi:10.3389/fmicb.2018.02908)

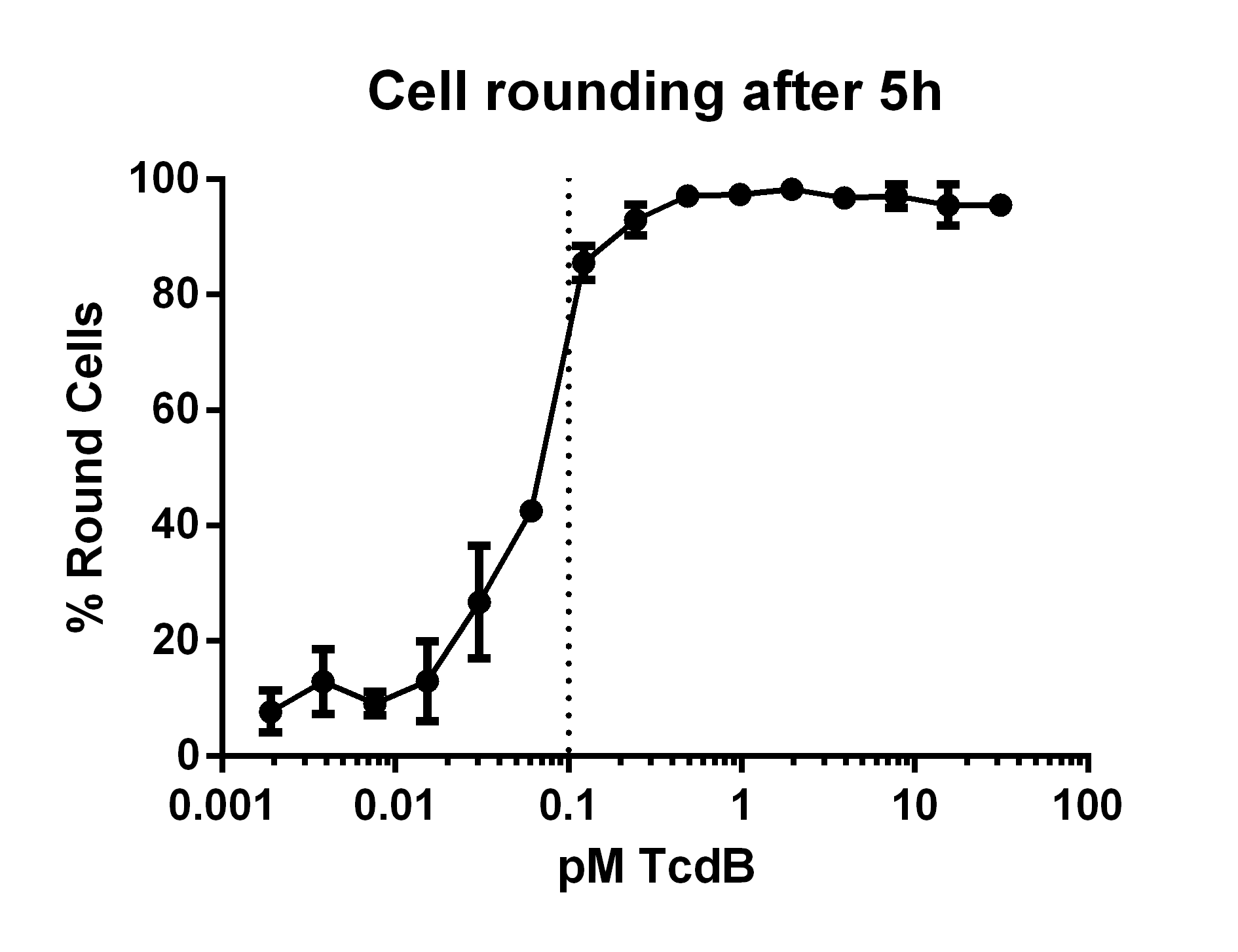

Supplement: Supplementary file 2 [file Image_1.TIF]

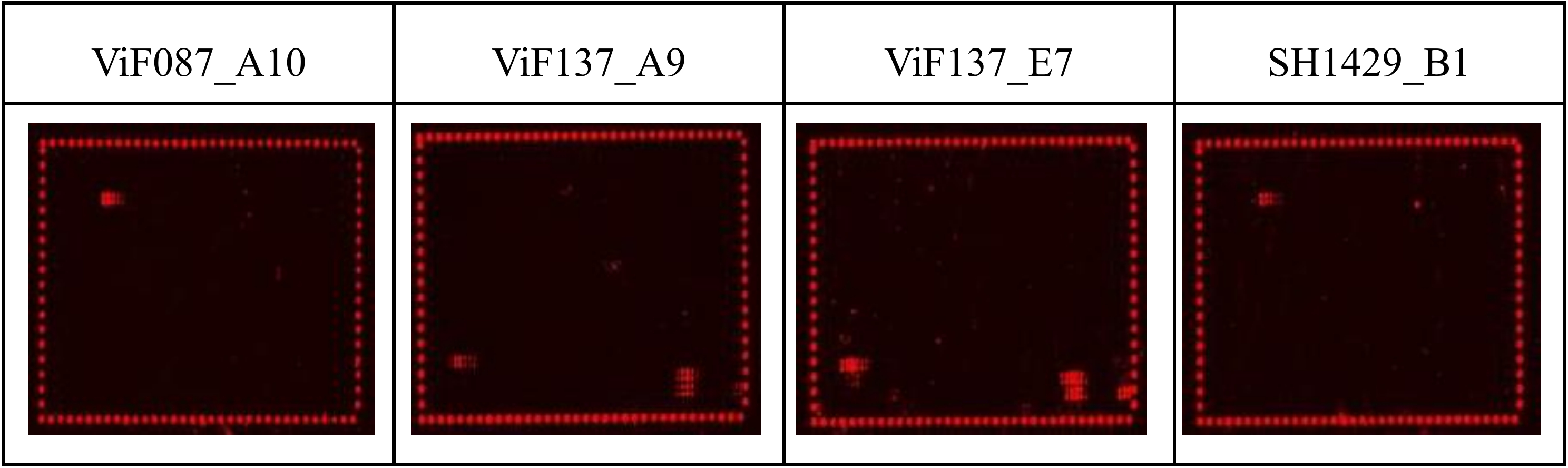

Supplement: Supplementary file 3 [file Image_2.JPEG]

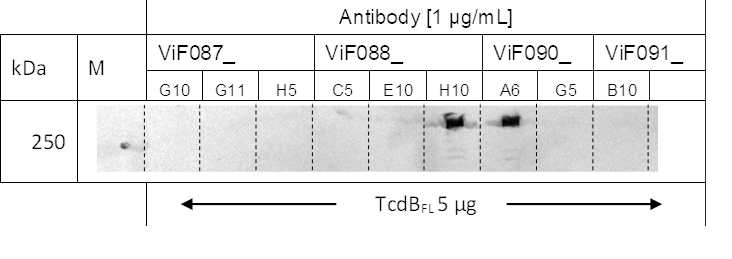

Supplement: Supplementary file 4 [file Image_3.JPEG]
